# Severity of allergic rhinitis impacts sleep and anxiety: results from a large Spanish cohort

**DOI:** 10.1186/s13601-018-0212-0

**Published:** 2018-07-09

**Authors:** R. Muñoz-Cano, P. Ribó, G. Araujo, E. Giralt, J. Sanchez-Lopez, A. Valero

**Affiliations:** 1 Allergy Unit, Pneumology Department, Hospital Clinic, Universitat de Barcelona, ARADyAL, Barcelona, Spain; 2 Institut d’Investigacions Biomèdiques August Pi i Sunyer, IDIBAPS, Barcelona, Spain; 3 Allergy Unit, Pneumology Department, Hospital Clinic, Universitat de Barcelona, CIBERES, Barcelona, Spain; 4 Grupo SANED, Barcelona, Spain; 5 Laboratorios LETI SL, Barcelona, Spain

**Keywords:** Anxiety, Depression, Allergic rhinitis, Sleep

## Abstract

**Background:**

Allergic rhinitis (AR) is a highly prevalent disease that generates high social and health care costs and also has a significant effect on quality of life and quality of sleep. It has also been related to some psychological disorders like anxiety or depression.

**Objective:**

To evaluate anxiety, depression, and quality of sleep and life alteration in a group of patients with perennial AR compared to a group of seasonal AR patients.

**Methods:**

Six-hundred seventy adults (> 18 years) with perennial and seasonal AR were recruited consecutively in 47 centers in Spain. Individuals were grouped in “Perennial” and “Seasonal” according to the seasonality of their symptoms. Anxiety, depression, sleep quality and health related quality of life were evaluated using the Hospital Anxiety and Depression Scale, Medical Outcomes Study Sleep Scale (MOS Sleep Scale) and the Health-related quality of life questionnaire ESPRINT-15, respectively. Both groups of patients were evaluated in and out of the pollen season.

**Results:**

AR symptoms are related to worse quality of life and more anxiety and depression symptoms. Indeed, symptom severity also correlates with worse outcomes (quality of life, sleep and depression/anxiety) regardless allergen seasonality. Symptoms severity, compared with seasonality and persistence, is the most important factor related with more anxiety and depression and poor sleep. However, symptoms severity, persistence and seasonality are independently affecting the quality of life in patients with AR.

**Conclusions:**

Although AR symptoms have a great impact on depression and anxiety symptoms, quality of life and quality of sleep in all AR patients, as expected, individuals with more severe AR seem to suffer more intensely their effects.

**Electronic supplementary material:**

The online version of this article (10.1186/s13601-018-0212-0) contains supplementary material, which is available to authorized users.

## Background

Allergic rhinitis (AR) is a highly prevalent disease that generates high social and health care costs [1] and also has a significant effect on quality of life (QoL) [2–4]. AR accounts for 55.5% of all cases in Spanish allergy clinics consultations [5], and it is known to alter patients’ social life, nighttime rest and inducing daytime drowsiness [6]. Consequently, its adverse impact upon school and work performance has been described [7, 8]. In addition, AR is associated to several disorders with a strong socioeconomic impact such as asthma and rhinosinusitis [1, 9], with the subsequently added impact upon health-related quality of life (HRQL). Olfactory dysfunction is also a very important symptom in patients with AR, and it has been considered as a key contributor to quality of life; its loss is associated to a decrease in both food/drink enjoyment and a social competence [10].

Sleep quality is also altered in patients with AR, and its severity correlates with a poorer sleep quality [2]. AR can impair nocturnal sleep through a mechanical mechanism related to nasal obstruction, snoring and apnea/hypopnea [11, 12]. Sleep impairment has subsequent effects on daytime performance and HRQL. Moreover, the inflammatory cytokines released during allergic reactions have been related to the suppression of both rapid eyes movement (REM) and no-REM phase [13, 14].

A positive association between allergies and psychological disorders has been stablished demonstrated in several studies. Patten et al. [15] provided evidence of AR patients having a higher rate of panic disorder and social phobia (OR 1.7 and 1.3, respectively) than healthy individuals, as well as major depression (OR 1.5). Likewise, Cuffel et al. [16] analyzed 850.000 individuals and found that anxiety symptoms were 1.4 times higher in individuals with AR versus healthy controls. Indeed, depression diagnosis was 1.7 times more frequent. Several hypotheses have been formulated to explain the association between AR and psychological disorders. The effects of both anxiety and rhinitis on immunity, affecting cytokine production/release, may account for this relationship. On the other hand, the impairing effects of nasal obstruction on sleep have a negative effect on psychiatric symptoms. Finally, a possible shared genetic risk between both allergy and depression has been postulated [17].

The duration of allergen exposure (i.e. perennial vs seasonal) may have a differential impact on sleep quality, anxiety and QoL [18]. A perennial exposure, with persistent and perennial symptoms, may affect these parameters more deeply than seasonal exposure. Our objective is to compare anxiety and depression levels, sleep disturbance and quality of life in a group of patients with perennial AR with a group of seasonal AR patients.

## Methods

### Patient selection and study design

**Fig. 1:**
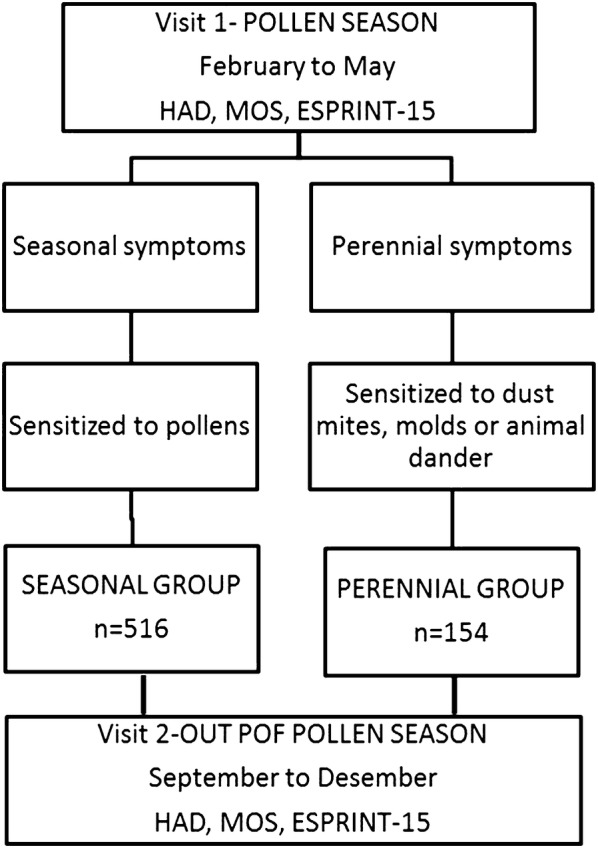
Study flow chart. HAD: Hospital Anxiety and Depression Scale; MOS: Medial Outcomes Study Sleep Scale; ESPRINT-15: health-related quality of life in adults with allergic rhinitis

Six-hundred seventy adults (> 18 years) with perennial and seasonal AR were recruited consecutively in 47 centers in Spain. Individuals were grouped in “Perennial” and “Seasonal” groups according to the seasonality of their symptoms and allergic sensitization. Skin prick test with dust mite, molds, dog and cat dander and pollens (plane tree, olive tree, grass, wall pellitory, cypress) were performed in all patients. Patients in the “*perennial group*” had symptoms all over the year and were sensitized to dust mites, molds and animal dander. Those with symptoms limited to pollen season, sensitized to pollen (cypress, grass, olive tree, plane tree and wall pellitory) and symptomatic at visit 1, were included in the “*seasonal group*”. Both groups were evaluated during the pollen season (visit 1 from February to May) and out of the pollen season (visit 2 from September to December). Pregnant women, individuals with psychiatric disorders, patients treated with antidepressants or anxiolytics, and individuals receiving immunotherapy were excluded (see Fig. 1).

Informed consent was obtained from all participating subjects. The study was approved by the Local Ethics Committee of Hospital Clinic, (Barcelona, Spain).

### Variables

Anxiety, depression, sleep quality and health related quality of life were evaluated using validated questionnaires: the Hospital Anxiety and Depression Scale (HAD) [19, 20], Medical Outcomes Study Sleep Scale (MOS Sleep Scale) [21, 22] and the Health-related quality of life questionnaire ESPRINT-15 [4, 23], respectively.

HAD is a self-administered 14 items questionnaire with a total score from 0 to 42. Seven of these items measure anxiety (score from 0 to 21) and the other seven items measure depression (from 0 to 21). Scores from 11 to 21 indicate a clinical problem, from 8 to 10 the results are not conclusive, and from 0 to 7 are in the range of normality. Participants are asked to work through each question indicating the extent to which they have experienced a particular symptom or state over the past week.

MOS Sleep Scale is a self-administered 12‐item questionnaire developed to provide a concise assessment of 6 important dimensions of sleep, including initiation, maintenance, respiratory problems, quantity, perceived adequacy and somnolence. Two sleep problems indexes (sleep index I and II) summarizing information across all these items were also used. Quality of sleep is expressed on a numeric rating scale ranging from 0 (“best possible sleep”) to 10 (“worst possible sleep”). Participants are asked to work through each question indicating the extent to which they have experienced a particular symptom or state over the past week.

ESPRINT-15 is a specific and validated instrument to measure health-related quality of life in adult patients with AR, which was first validated for use in Spanish-speaking populations. This questionnaire has shown good reliability, validity, and sensitivity to change. It has also proved easy to use and administer. This self-administered questionnaire contains 15 items distributed within the following dimensions: symptoms (5 items), daily activities (3 items), sleep (3 items), psychological impact (3 items), and general health (1 item). An overall score and a score for each dimension are obtained. The overall score and the dimensional scores range from 0 (no impact on HRQOL) to 6 (maximum impact on HRQOL). Participants are asked to work through each question indicating the extent to which they have experienced a particular symptom or state over the past 2 weeks.

Co-morbidities as rhinitis and asthma and their severity using ARIA and GINA guidelines respectively, were recorded.

### Statistics

Qualitative variables were described using frequencies and quantitative variables by means of centralization and dispersion measures. For comparisons, Fisher’s exact test for categorical variables or a Student’s *t* test were used. For comparisons with three or more categories an ANOVA test was implemented. To evaluate the effect of symptoms severity, seasonality and persistence in the variables, a multivariate analysis was performed. Quantitative variables were evaluated using a multiple lineal regression model and post-oc differences were evaluated using Tuckey test. Qualitative variables were evaluated using a logistic regression. A value of *p* < 0.05 was considered significant.

## Results

### Study population

Among the 670 recruited patients, 516 corresponded to the *seasonal group* and 154 to the *perennial group*. No differences were found between groups, except for conjunctivitis that was more frequently observed in *seasonal patients* than in *perennial patients* (37.8 vs. 16.2%; *p* < 0.001) (Table 1).

**Table 1:** Demographics, sensitization profile and clinical manifestations

	Perennial (n = 154)	Seasonal (n = 516)
Age (years) (mean ± SD)	34.2 ± 11.6	34.7 ± 10.9
Gender (female %)	62.5	57.8
Rhinitis (%)		
Persistent	67.5	63.6
Intermittent	32.5	36.4
Mild	20.1	19.2
Moderate	63	56.4
Severe	16.9	24.4
Co-morbidities (%)		
Conjunctivitis	16.2*	37.8
Asthma	40.9	41.5
Intermittent	58.3	49
Mild persistent	21.7	30
Moderate persistent	18.3	19
Severe persistent	1.7	1.9
Sensitization profile (%)		
Dust mites	96.1	34.8
Molds	3.9	5.2
Animal dander	20.1	20.1
Grass pollen	0	70.7
Olive tree pollen	0	51
Cypress pollen	0	20.5
Plane tree	0	15.9
Wall pellitory pollen	0	10.8

Asthma and rhinitis were classified using GINA and modified-ARIA classification respectively. *p < 0.01

### Effect of allergen exposure in AR patients with perennial symptoms

To assess the effect of allergen exposure on anxiety, depression and quality of sleep and life, results from both *perennial group* and *seasonal group* were compared out of the pollen season. AR patients with seasonal symptoms were asymptomatic out of the pollen season; therefore they were used as a control group. No differences were observed in and out of pollen season in any variable of the study in patients with *perennial symptoms.*

**Fig. 2:**
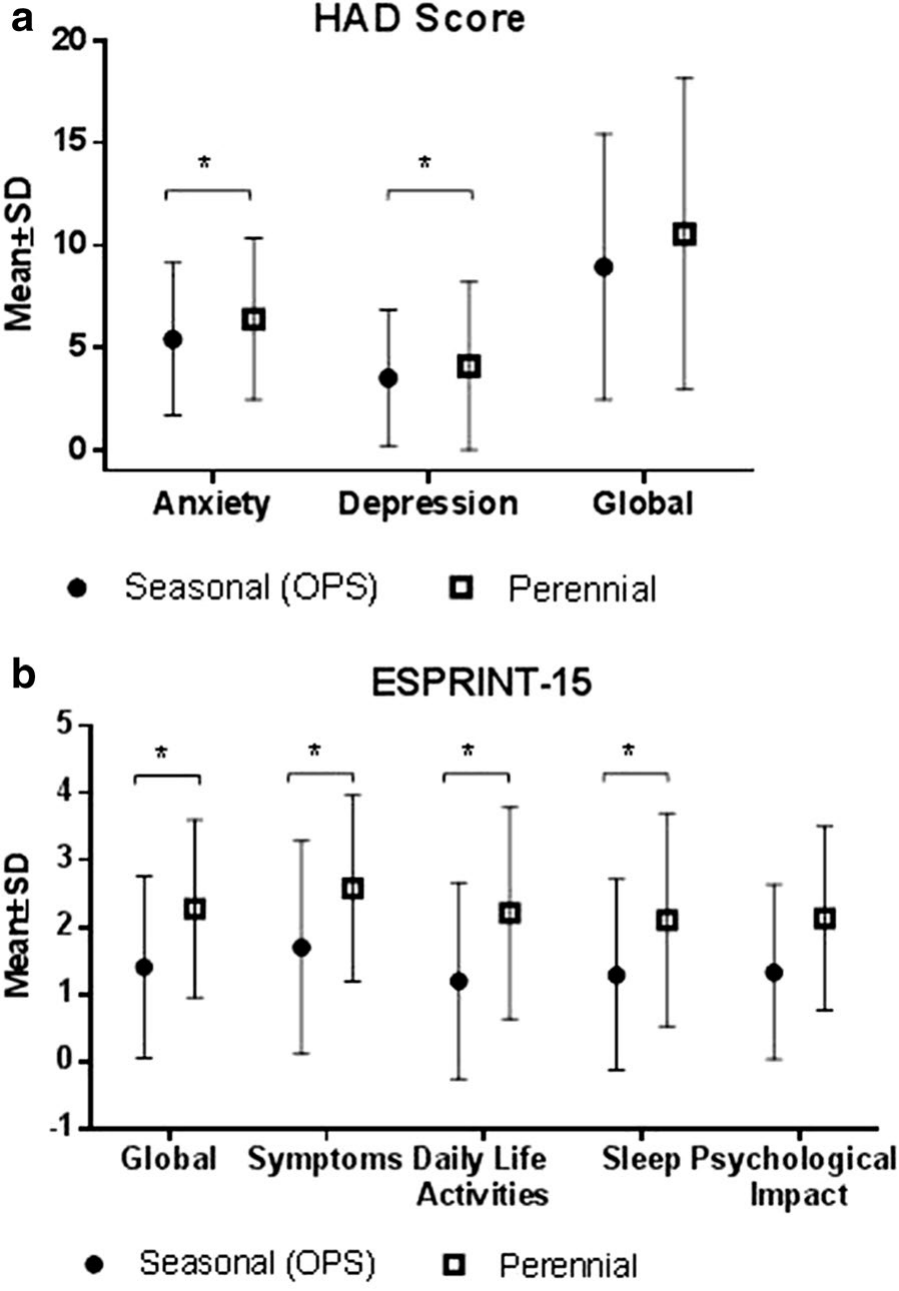
**a** Impact of allergen exposure on anxiety and depression measured by HAD scale in patients with perennial symptoms compared with seasonal allergic rhinitis patients out of pollen season. **b** Impact of allergen exposure on quality of life measured by ESPRINT-15 in patients with perennial symptoms compared with seasonal allergic rhinitis patients out of pollen season. OPS: out of pollen season. HAD: Hospital Anxiety and Depression Scale. ESPRINT-15: Health-related quality of life questionnaire. *p < 0.05

Patients with *perennial symptoms* scored higher for anxiety (6.4 ± 3.9 vs. 5.4 ± 3.7; *p* = 0.03) and depression (4.1 ± 4.1 vs. 3.5 ± 3.3; *p* = 0.17) compared to asymptomatic individuals (Fig. 2a). However, the total number of patients diagnosed with anxiety (HAD Anxiety > 11; 17.6% perennial vs. 12.1% seasonal; *p* = 0.17) or depression (HAD Depression > 11; 9.9% perennial vs. 5% seasonal; *p* = 0.15) was not different.

Patients in the *perennial group* had a quality of sleep similar to the asymptomatic individuals, and they only scored higher in the dimension “sleep shortness of breath” (24.2 ± 26.9 vs. 15.6 ± 20.7; *p* = 0.0016). A similar number of patients had a suboptimal sleep measured by MOS in both groups (36 vs. 29.9%, *p* > 0.05).

Patients with AR in the *perennial group* had a worse quality of life (higher score in all dimensions) measured by ESPRINT-15 questionnaire compared to the asymptomatic control group (Fig. 2b). Global ESPRINT-15 score in *perennial group* was 2.3 ± 1.3 and 1.4 ± 1.3 in the control group (*p* < 0.05).

Symptoms persistence and severity were related with worse outcomes (anxiety, depression, sleep and quality of life) in perennial AR individuals (Table 2).

**Table 2:** Anxiety, depression, quality of life and quality of sleep in AR patients with perennial and seasonal symptoms depending on symptoms severity and persistence

	Persistent	Intermitent	*p* value	Mild	Moderate	Severe	*p* value
	n = 104	n = 50		n = 31	n = 97	n = 26	
Perennial							
HAD anxiety	6.4 ± 3.9	5.3 ± 3.7	**^c^	4.5 ± 3.8	6 ± 3.6	7.3 ± 4	***^a^
HAD depression	4.2 ± 3.6	3.5 ± 3.4	*^c^	3.4 ± 3.5	3.6 ± 3.3	5.3 ± 4	***^a^
HAD anxiety > 11	17.2%	11%	ns^b^	9.4%	13. %	23.3%	**^a^
HAD depression > 11	31%	13%	ns^b^	6.3%	4.1%	13.2%	***^b^
MOS Sleep index I	64.8 ± 18.8	71.9 ± 17.1	***^c^	76.4 ± 16.6	68.2 ± 17.6	57.4 ± 17.8	***^a^
MOS Sleep index II	65.5 ± 18	72.6 ± 16.7	***^c^	77.3 ± 15.9	68.7 ± 16.9	58.6 ± 17.2	***^a^
MOS suboptimal sleep	40%	29.7%	*^b^	36.4%	33.5%	43.4%	ns^b^
ESPRINT global	2.5 ± 1.4	1.3 ± 1.2	***^c^	0.8 ± 0.9	2.2 ± 1.1	2.9 ± 1.6	***^a^

	n = 328	n = 188		n = 99	n = 291	n = 126	
Seasonal							
HAD anxiety	6.4 ± 3.8	6.2 ± 3.8	ns	6 ± 4.1	5.9 ± 3.5	7.3 ± 3.9	*^a^
HAD depression	4.3 ± 3.4	3.6 ± 3.3	ns	4.3 ± 3.9	3.4 ± 3	5.5 ± 3.6	***^a^
HAD anxiety > 11	16.1%	16.1%	ns	20.8%	11.4%	24.1%	ns^b^
HAD depression > 11	4.7%	4.9%	ns	8.7%	1.8%	9.5%	*^b^
MOS Sleep index I	63.2 ± 19.2	67.9 ± 17.3	ns	69.2 ± 18.4	67.7 ± 18.2	56.2 ± 18.1	***^a^
MOS Sleep index II	63.9 ± 18.1	67.8 ± 17.4	ns	70 ± 17.3	67.9 ± 17.4	57 ± 18.2	***^a^
MOS suboptimal sleep	43.2%	35.5%	ns	41.7%	37.7%	48.8%	ns^b^
ESPRINT global	2.8 ± 1.3	1.8 ± 1.1	***^c^	1.3 ± 0.9	2.4 ± 1	3.3 ± 1.4	***^a^

Values expressed as mean ± SD. Comparisons between severity degrees (mild vs. moderate vs. severe) and symptoms persistence (intermittent vs. persistent) in both perennial AR and seasonal AR were made using ^a^ANOVA; ^b^Fisher’s exact test and ^c^*t* test. *p* value ≤ *0.01, *0.001, ***0.000001

Rhinitis severity was classified using modified-ARIA (Ref. [30]); HAD: Hospital Anxiety and Depression Scale (Ref. [19]), MOS: Medical Outcomes Study Sleep Scale (ref. [21]); ESPRINT-15: Health-related quality of life questionnaire (Ref. [4, 23])

### Effect of allergen exposure in AR patients with seasonal symptoms

To assess the effect of allergen exposure on anxiety, depression and quality of sleep, we compared the *seasonal group* patients in and out of pollen season.

**Fig. 3:**
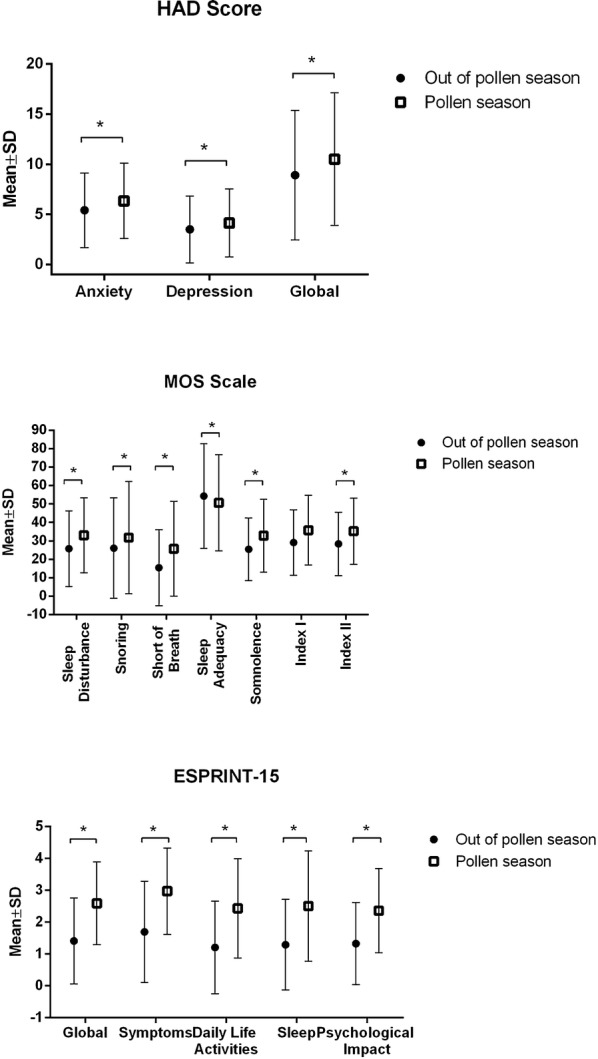
**a** Impact of allergen exposure on anxiety and depression measured by HAD scale in allergic rhinitis patients with seasonal symptoms in and out of pollen season. **b** Impact of allergen exposure on quality of sleep measured by MOS scale in allergic rhinitis patients with seasonal symptoms in and out of pollen season. **c** Impact of allergen exposure on quality of life measured by ESPRINT-15 in allergic rhinitis patients with seasonal symptoms in and out of pollen season. ESPRINT-15: Health-related quality of life questionnaire. HAD: Hospital Anxiety and Depression Scale. MOS: Medical Outcomes Study Sleep Scale. *p < 0.05

Patients with *seasonal symptoms* had higher global HAD scores during pollen season than out of pollen season (8.9 ± 6.5 vs. 10.5 ± 6.6; *p* < 0.05). Both anxiety and depression scores were higher during pollen season (6.3 ± 3.8 vs. 5.4 ± 3.7; *p* < 0.05; 4.1 ± 3.4 vs. 3.5 ± 3.3; *p* < 0.05, respectively) (Fig. 3a). However, the number of patients diagnosed with anxiety or depression (HAD > 11) was not different in or out of pollen season (16 vs. 12%; *p* > 0.05; 4.8 vs. 5%; *p* > 0.05 respectively).

Similarly, MOS Sleep indexes were higher during pollen season reflecting a poorer quality of sleep with more somnolence, apneas and snoring (Fig. 3b). Up to 41.5% of patients had a suboptimal sleep during pollen season and 30% out of pollen season (*p* = 0.007).

Quality of life measured using ESPRINT-15 questionnaire was more affected during pollen season in all 5 dimensions, including global score, symptoms, daily life activities, sleep and psychological disturbance. ESPRINT-15 global score was also higher during pollen season, meaning worse quality of life (2.6 ± 1.3 vs. 1.4 ± 1.3 *p* < 0.05) (Fig. 3c).

In the seasonal group, symptoms severity but not persistence was related with worse outcomes (anxiety, depression, sleep and quality of life) (Table 2).

### Effect of allergen exposure duration, symptoms seasonality and severity on AR patients

To assess the effect of allergen exposure duration and symptoms seasonality and severity on anxiety, quality of the sleep and quality of life, we compared both groups when symptomatic. That is during pollen season in seasonal group and together in and out of pollen season in perennial group.

When comparing both groups and adjusting for symptoms severity, seasonality and persistence, we found that severity is the most important factor related with worse outcomes in most of the evaluated parameters.

Both symptoms severity and seasonality were independently related with worse HAD scores. Perennial group and patients with more severe symptoms had worse outcomes. In that way, the risk of depression (HAD > 11) was increased 3.3-fold in perennial AR patients compared with seasonal AR, and 2.7-fold in severe AR compared with mild and moderate AR individuals. However, only severity was significantly related with anxiety diagnosis (HAD > 11). Severe AR patients had an increased risk (2.6-fold) of developing anxiety (HAD > 11) compared to moderate or mild AR individuals, independently of their symptoms seasonality or persistence (Table 3).

**Table 3:** Effect of symptoms seasonality, persistence and severity on anxiety, sleep and quality of life in AR patients-multivariate analysis

	Perennial versus seasonal *p* value	Mild versus moderate versus severe *p* value	Intermittent versus persistent *p* value
HAD global	0.02	< 0.0001	ns
HAD anxiety	ns	< 0.0001	ns
HAD depression	0.03	< 0.0001	ns
MOS Sleep index I	ns	< 0.0001	ns
ESPRINT	0.005	< 0.0001	< 0.0001

The effect of symptoms severity, seasonality and persistence in each variable was evaluated using a multivariate analysis. Quantitative variables were evaluated using a multiple lineal regression model and post-oc differences were evaluated using Tuckey test. A value of *p* < 0.05 was considered significant

Symptoms severity and persistence were independently associated with suboptimal sleep, with patients with moderate and persistent AR symptoms having an increased risk (1.6-fold and twofold respectively) of having trouble sleeping (Table 3).

Finally, all three variables, persistence, severity and seasonality were independently related with worse quality of life measured using ESPRINT. Patients with severe AR, perennial and persistent symptoms had a worse quality of life compared with moderate o mild AR, seasonal and intermittent symptoms respectively (Table 3).

### Effect of the comorbidities on anxiety, depression, quality of sleep and quality of life in patients with AR

The impact of the most common comorbidities in AR, such as asthma and conjunctivitis, on anxiety, depression, quality of sleep and quality of life were evaluated during the symptomatic period of each group.

In *perennial AR patients*, neither asthma nor conjunctivitis were related to worse outcomes: no statistically significant differences were detected in any of the MOS scale domains, HAD depression/anxiety scores or diagnosis or quality of life (data not shown). However, in *seasonal AR patients,* asthma was related with worse sleep quality, mainly in “sleep disturbance” and “sleep shortness of breath”. Those patients also scored higher in HAD depression and anxiety, and more patients were diagnosed with anxiety/depression compared to non-asthmatic patients. However, asthmatics’ quality of life measured by ESPRINT-15 questionnaire was not statistically different compared to those without asthma (Table 4). On the other hand, conjunctivitis was not related to worse results in any of the measured outcomes (data not shown).

**Table 4:** Effect of asthma on anxiety, depression, quality of life and quality of sleep in seasonal allergic rhinitis patients

	Seasonal AR + ASTHMA N = 210	Seasonal AR without Asthma n = 306	*p* value
HAD anxiety	6.9 ± 3.9	5.9 ± 3.6	*^a^
HAD anxiety > 11	20.3%	12.7%	*^b^
HAD depression	4.8 ± 3.8	3.6 ± 3.1	**^a^
HAD depression > 11	9%	1.3%	**^b^
ESPRINT Global	2.6 ± 1.3	2.6 ± 1.3	ns^b^
MOS-Sleep disturbance	63.4 ± 21	69.9 ± 19.4	**^a^
MOS-Sleep shortness of breath	70.5 ± 25.6	77.5 ± 25.5	*^a^
MOS Sleep index I	61.6 ± 19.4	66.5 ± 18.3	*^a^
MOS Sleep index II	61.9 ± 18.5	67.1 ± 17.3	*^a^
MOS Suboptimal sleep	46%	37.7%	ns^b^

Values expressed as mean ± SD. Comparison between groups (seasonal AR with vs without asthma) using ^a^*t* test and ^b^Fisher’s exact test. *p ≤ 0.05; **p ≤ 0.01; ns: not significant

### Correlation between the studied variables

We calculated the correlation between the evaluated variables (anxiety, depression, quality of sleep and quality of life) to study the impact on each other. In both groups, patients with a worse score in quality of sleep had significantly higher scores in HAD depression and anxiety and had significantly worse quality of life measured by ESPRINT-15. No differences in the correlations were observed when comparing *seasonal* and *perennial patients* (Additional file 1: Suppl Table 1).

## Discussion

This is the first study that has evaluated anxiety and depression, using validated questionnaires, in patients with seasonal and perennial AR. We have found that AR symptoms are related with worse quality of life and more anxiety and depression symptoms. Indeed, symptoms severity also correlates with worse outcomes (quality of life, sleep and depression/anxiety) regardless allergen seasonality. Interestingly asthma is associated with worse outcomes exclusively in individuals with seasonal symptoms.

In our study, individuals with AR symptoms had worse QoL than asymptomatic ones. Previous studies using different questionnaires (RQLQ, ESPRINT-15) have also revealed the impact of AR on QoL [24]. Indeed, the significant correlation found between QoL and AR severity made possible to differentiate between moderate and severe AR in the modified version of ARIA guideline [3, 4, 25]. We have found that symptomatic AR patients scored higher on anxiety and depression tests than asymptomatic individuals. However, there was no more anxiety/depression diagnosis. Previous works have shown that anxiety symptoms are more frequent in patients with allergies. Cuffel et al. [16] examined the healthcare claims of more than 5000 individuals and found that anxiety symptoms were 1.4 times higher and depression diagnosis was 1.7 times higher in individuals with allergies versus those without allergies. Likewise, in their examination of more than 12,000 individuals, Patten et al. [15] determined that major depression was more frequent in individuals with allergies (OR 1.5). Finally, we found a differential effect of AR symptoms on quality of sleep in perennial AR compared to seasonal AR. Whereas seasonal AR patients had worse quality of sleep when symptomatic, that was not the case of the individuals with perennial AR. Previous studies have shown that patients with AR have an impaired sleep and this correlates with disease severity, either in intermittent or persistent AR [2, 11, 12, 26]. In our study, although no sleep disturbances were found in perennial AR using MOS scale, the ESPRINT-15 domain referring to sleep was significantly affected. The differences between the tests used to evaluate sleep impairment in all those studies may account for the different results. Sleep disturbances make patients more susceptible to psychological consequences, as fatigue, irritability, anxiety and depression, which in the end can result in daytime somnolence and finally contribute to QoL impairment. However, the use of antihistamines to treat AR may also contribute to patient somnolence and cannot be ruled out [27].

We have observed that all three, symptoms persistence, severity and seasonality are affecting independently the quality of life of AR patients. Thus, patients with persistent, severe and perennial AR have worse quality of life measured using ESPRINT. However, other studies did not find differences in the QoL of patients with AR sensitized to perennial and seasonal allergens [28]. A previous study evaluated the psychological status of patients with seasonal and perennial AR [29] and the influence of type, duration, and severity of rhinitis on the psychiatric evaluation. Forty-one patients with seasonal and perennial AR and 36 healthy control subjects were enrolled in the study but differences between seasonal and perennial AR patients were not significant. However, severity was positively correlated with anxiety, whereas negatively correlated with the score of satisfaction with the life scale. Actually, a previous study evaluating severity and duration of AR in 3052 patients concluded that the impact that AR severity had on quality of life was more significant than was the duration (intermittent vs persistent) of the disease; 80% of the patients with the severe forms reported impairment in their activities due to the disease, compared with only 40% of those with mild forms [31].

Interestingly, several studies have shown the effect of stress not only in the exacerbation of allergy symptoms but in the development of both asthma and rhinitis. Wright et al. found that, in children predisposed to asthma, increased stress in early childhood was associated with an atopic immune profile [32]. Kilpelainen et al. observed that concomitant parental and personal conflicts increased the risk of asthma (OR 1.72, 95% CI 1.10–2.69) when adjusted by parental asthma, education and passive smoking. However, asthma and atopic dermatitis but not AR, were related to excess of stressful life events [33]. Finally, Rod et al. showed that perceived stress was associated with atopic disorders in a dose-dependent manner. High stress was associated with higher risk of allergic rhinitis (OR 1.64; 95% CI 0.99–2.72), asthma incidence (OR 2.32; 95% CI 1.47–3.65) and first-time asthma hospitalization (HR 2.01; 95% CI 1.41–2.86) among others [34].

Co-morbidities have shown to play a role in AR-related quality of life. However, in our study frequent co-morbidities such as asthma or conjunctivitis had a low impact on the studied outcomes. Conjunctivitis had no effect in any of the tested variables in any group. Asthma was related with higher scores for anxiety/depression and impairment of quality of sleep, but exclusively in seasonal AR. Although the acuteness of asthma symptoms appearance during pollen season may explain their impact only in seasonal AR, whether asthma symptoms were present exclusively during pollen season was not collected. A previous study showed that AR had a more detrimental impact on QoL in patients with comorbid asthma than in patients without asthma, and it was even more important in severe asthma [35]. On the other hand, Colas et al. [2.] found that asthma was related with worse quality of sleep only in moderate AR, but not in mild or severe AR. Another study of 524 patients with intermittent and persistent AR showed that the effect of AR on the psychological profile of patients was independent of age and gender, but it was significantly associated with comorbid asthma [36]. Slattery et al. described that anxiety was associated with asthma and AR, and having both asthma and AR strengthened the association compared to having either disorder alone [37].

However, the relationship between asthma and psychological disorders has been established regardless of AR presence. Mancuso et al. [38] demonstrated that 45% of the 230 asthmatic patients evaluated were positive for depressive symptoms and that these patients reported worse health-related quality of life than asthma patients with similar disease activity but fewer depressive symptoms. Other authors [39] found significant differences in somatization, obsessive–compulsive, depression, and anxiety among patients with a history of eczema or asthma compared with patients who did not have such a history. Collectively, all these findings suggest that asthma may exert an independent effect on the psychological status of patients with AR and show the importance of assessing and treating all AR patients for asthma and for other common chronic comorbid conditions.

This study has some limitations. Patients in the “seasonal group” were recruited at visit 1 (during pollen season) only if they had symptoms (inclusion criteria). Although all patients were symptomatic, at visit 1 some patients may have had symptoms longer than others and this could affect their impact on anxiety, depression, quality of sleep and life. Another limitation is that we did not consider the effect of disease control on the studied variables; uncontrolled AR may have greater impact on anxiety/depression or sleep quality than a well-controlled disease. Finally, we did not use healthy individuals as control group and we used seasonal AR patients out of pollen season instead. Probably, the comparison of seasonal and perennial AR patients with a real control population would be indicative of the real anxious/depressive background of those patients.

In conclusion, AR symptoms have a great impact on quality of life, quality of sleep, and depression and anxiety symptoms in all AR patients. However, individuals with perennial and severe symptoms seem to suffer more intensely their effects.

## Additional file

**Additional file 1: Suppl. Table 1.** Correlation between anxiety, depression, quality of sleep and quality of life in perennial and seasonal groups.

## Abbreviations

AR: allergic rhinitis
HAD: Hospital Anxiety and Depression Scale
HRQoL: health-related quality of life
MOS: Medical Outcomes Study Sleep Scale
QoL: quality of life
