# Supplementary material for: Severity of allergic rhinitis impacts sleep and anxiety: results from a large Spanish cohort
Source: Clin Transl Allergy. 2018 Jul 9;8:23. doi: 10.1186/s13601-018-0212-0 (PMC6036679; doi:10.1186/s13601-018-0212-0)
Supplement: Supplementary file 1 — Additional file 1: Suppl. Table 1. Correlation between anxiety, depression, quality of sleep and quality of life in perennial and seasonal groups. [file 13601_2018_212_MOESM1_ESM.docx]

**Suppl. Table 1. Correlation between anxiety, depression, quality of sleep and quality of life in perennial and seasonal groups.**

|  |  | **MOS** | **ESPRINT global** |
| --- | --- | --- | --- |
| **Perennial** | **HAD anxiety** | -0.49* | 0.44* |
|  | **HAD depression** | -0.44* | 0.3* |
|  | **MOS Sleep I** |  | -0.5* |
|  | **MOS Sleep II** |  | -0.54* |
| **Seasonal** | **HAD anxiety** | -0.55* | 0.24* |
|  | **HAD depression** | -0.55* | 0.16* |
|  | **MOS Sleep I** |  | -0.51* |
|  | **MOS Sleep II** |  | -0.53* |

Correlations using Spearman test. Values expressed as Rho. *p values ≤0.001
